# The Chaperonin GroESL Facilitates Caulobacter crescentus Cell Division by Supporting the Functions of the Z-Ring Regulators FtsA and FzlA

**DOI:** 10.1128/mBio.03564-20

**Published:** 2021-05-04

**Authors:** Kristen Schroeder, Kristina Heinrich, Ines Neuwirth, Kristina Jonas

**Affiliations:** a Science for Life Laboratory and Department of Molecular Biosciences, The Wenner-Gren Institute, Stockholm University, Stockholm, Sweden; Max Planck Institute for Terrestrial Microbiology

**Keywords:** FtsA, FzlA, GroEL, bacterial cell division, chaperonin, peptidoglycan, protein folding, actin-like proteins

## Abstract

The highly conserved chaperonin GroESL performs a crucial role in protein folding; however, the essential cellular pathways that rely on this chaperone are underexplored. Loss of GroESL leads to severe septation defects in diverse bacteria, suggesting the folding function of GroESL may be integrated with the bacterial cell cycle at the point of cell division. Here, we describe new connections between GroESL and the bacterial cell cycle using the model organism Caulobacter crescentus. Using a proteomics approach, we identify candidate GroESL client proteins that become insoluble or are degraded specifically when GroESL folding is insufficient, revealing several essential proteins that participate in cell division and peptidoglycan biosynthesis. We demonstrate that other cell cycle events, such as DNA replication and chromosome segregation, are able to continue when GroESL folding is insufficient. We further find that deficiency of two FtsZ-interacting proteins, the bacterial actin homologue FtsA and the constriction regulator FzlA, mediate the GroESL-dependent block in cell division. Our data show that sufficient GroESL is required to maintain normal dynamics of the FtsZ scaffold and divisome functionality in C. crescentus. In addition to supporting divisome function, we show that GroESL is required to maintain the flow of peptidoglycan precursors into the growing cell wall. Linking a chaperone to cell division may be a conserved way to coordinate environmental and internal cues that signal when it is safe to divide.

## INTRODUCTION

All cells must monitor and adjust the vital processes of growth and division in response to external and internal environmental cues. Prokaryotic model organisms offer an accessible system to study how essential biological processes are regulated in response to these cellular and environmental signals. Molecular chaperones are ubiquitous proteins with high sequence conservation, and studying protein folding dynamics in bacterial systems has been crucial in understanding the fundamental processes that assist all organisms in building and maintaining functional proteins.

During biosynthesis, some proteins must overcome energy barriers in order to achieve their native fold (1). For these proteins, interaction with ATP-powered chaperones assists them in attaining a functional conformation on a biologically relevant time scale *in vivo* (1). This chaperone interaction is not limited to biosynthesis, as changes in intracellular conditions (e.g., temperature or oxidative stress, or the presence of toxic compounds) can destabilize folding of a wide array of proteins (2–4), which may then require refolding. To adjust chaperone folding capacity to these different folding demands, the expression of chaperone genes can be increased above basal levels through stress-responsive transcriptional control, for example through induction by the heat shock sigma factor (5). In this way, chaperone folding capacity is available for synthetic processes during optimal conditions and is increased to rescue misfolding of proteins during diverse stresses.

The majority of ATP-powered protein folding in prokaryotes is carried out by the highly conserved DnaK/J/GrpE-ClpB bichaperone system and the GroES/EL chaperonin machine (1), which are assisted in interacting with their client proteins by a network of less-conserved holdases, including small heat shock proteins and chaperedoxins (6). GroEL (Hsp60, Cpn60) is a heat shock protein that oligomerizes into a tetradecameric double ring structure with two central cavities that can capture unfolded proteins via their solvent-exposed hydrophobic residues (7). The GroES (Hsp10, Cpn10) co-chaperonin then binds as a lid over the GroEL-client complex, encapsulating client proteins and thus providing a segregated environment to assist with folding (7). While the number and arrangement of the *groES groEL* genes vary across bacteria, they are most often found in a single copy together in an operon that allows both for housekeeping expression, for example from a σ^70^-dependent promoter, as well as stress-responsive expression, for example from a σ^32^-dependent promoter or HrcA repressor sequences (5, 8, 9).

Despite detailed description of GroESL folding mechanics and a good understanding of the regulation of *groESL* transcription, comparably few studies exist examining the role of chaperonins in physiological processes. With the exception of some Mollicutes (10), GroESL is essential in all bacterial species investigated to date (9, 11–14). Several of these organisms exhibit a relationship between chaperonin availability and the cell cycle, as cell division is blocked when GroESL levels are reduced (13–15). During bacterial cell division, the presence, location, and quantity of many different classes of proteins, as well as the remodeling of the cell envelope, must be tightly controlled in order to successfully make two daughter cells. The regulation of these proteins can occur at the level of synthesis, degradation, conformation, localization, and activity (16, 17); however, the contribution of protein folding state and chaperone interactions to cell division is not yet well understood.

The most-well-studied bacterial chaperonin is that of Escherichia coli, where ∼250 proteins have been identified as interacting partners of GroESL (18, 19). Of these client proteins, 57 have been shown to obligately depend on GroESL for folding into the native state (classified as obligate, or type IV GroESL substrates), including 6 essential proteins (18–20). One of the identified essential obligate GroESL substrates is the cell division protein FtsE (19); however, while a functional deficit of this protein may contribute to the cell division defect reported during GroESL depletion in E. coli (21), the requirement for FtsE function can be bypassed by altering growth conditions, and it therefore remains unclear if FtsE is conditionally linked to GroESL in this organism. Chaperonin studies in other bacteria have identified a few proteins from the GroESL client protein pool (22–24), and it remains poorly understood how GroESL function impacts the cell cycle and other physiological processes in these organisms.

The model organism Caulobacter crescentus is a Gram-negative oligotrophic alphaproteobacterium with a dimorphic lifestyle that produces two morphologically distinct daughter cells (25). This asymmetric life cycle has made *Caulobacter* a powerful model for investigating events of the cell cycle. In particular, cell division has been well described in this organism, which has led to important advances being made in understanding the conserved mechanisms mediating cell division in bacteria (26, 27). Similarly to other bacteria, C. crescentus becomes filamentous when GroESL is depleted (15), indicating an involvement of GroESL in the cell cycle. However, the precise role of GroESL in *Caulobacter* cell cycle progression and cell division has not been studied so far.

In this study, we establish the connection between GroESL folding and cell division in C. crescentus. We have identified a subset of the proteome that changes solubility depending on the presence of the chaperonin, and show that several proteins involved in cell division and peptidoglycan (PG) biosynthesis become insoluble when GroESL folding capacity is reduced. Furthermore, we find that disruption of the division scaffold, or Z-ring, is associated with insufficient GroESL-mediated folding, and that increasing levels of the bacterial actin homologue FtsA or of the constriction regulator FzlA can delay the resulting filamentation phenotype observed during GroESL depletion. This underscores the importance of coordinating the process of cell division with protein biosynthesis, and further suggests a relationship between chaperonins and actin-like proteins in prokaryotes. Integrating chaperonin folding into cell division in this way may represent a way of coordinating environmental and internal cues that signal when it is safe to divide.

## RESULTS

### GroESL folding insufficiency results in filamentation.

**FIG 1:**
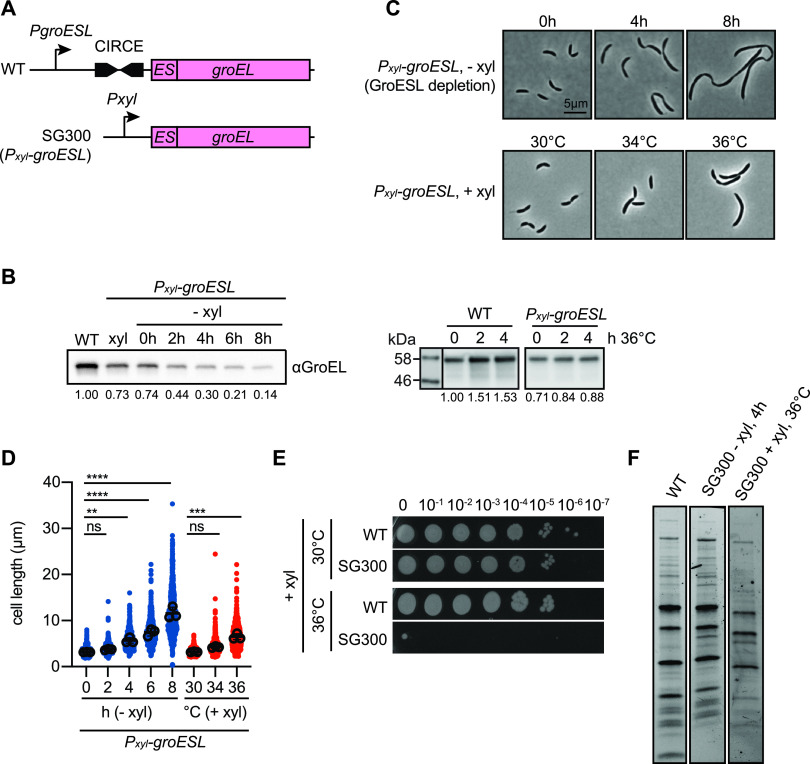
GroESL folding insufficiency results in cell filamentation. (A) Diagram of the GroESL locus in wild type (WT) and GroESL depletion (SG300, *P_xyl_-groESL*) strains. Wild type GroESL is regulated by the CIRCE element, as well as a σ^32^/σ^70^-dependent promoter, which has been replaced by the xylose-inducible promoter in strain SG300 (12). (B) Western blot of protein levels of GroESL during depletion (− xyl) and during exposure to 36°C. Quantifications of band intensities are an average of three (left panel) and two (right panel) biological replicates. (C) Phase-contrast microscopy showing the morphology of the GroESL depletion strain (*P_xyl_-groESL*) when grown in depleting conditions (− xyl), in non-depleting conditions (+ xyl), and at increased temperature. Cultures were depleted for the indicated time, or incubated for 4 h at the indicated temperatures prior to fixation and imaging. (D) Quantification of population cell lengths of the GroESL depletion strain (*P_xyl_-groESL*) during depletion (− xyl) or when grown under non-depleting conditions (+ xyl) at the indicated temperatures for 4 h (*n* = 200 for each of three biological replicates; ns, no significant difference; **, *P* < 0.01; ***, *P* < 0.001; ****, *P* < 0.0001). (E) Spot assay of wild type (WT) and the GroESL depletion strain (SG300) when grown under non-depleting conditions, incubated at the indicated growth temperatures. The GroESL-dependent temperature sensitivity is consistent with that previously reported (15). Xylose was included in all agar plates; plates were incubated for 2 to 3 days prior to imaging. (F) Coomassie staining of SDS-PAGE gel of insoluble fractions isolated from wild type and the GroESL depletion strain (SG300), grown either for 4 h in depleting conditions (− xyl), or incubated at 36°C for 4 h in non-depleting conditions (+ xyl).

As GroESL is essential in *Caulobacter* and cannot be deleted (12), we made use of the previously described C. crescentus strain SG300, where the regulatory region upstream of *groESL* is replaced by a xylose-inducible promoter (Fig. 1A) (12). When xylose is removed from the growth medium, GroESL is diluted from the growing culture over several hours (Fig. 1B). As GroESL levels decline, the demand for chaperonin-mediated folding exceeds what can be provided, resulting in the development of phenotypes associated with client protein misfolding (Fig. 1C). In C. crescentus, cells grow into long filaments featuring wide segments interspersed with irregular shallow constrictions (Fig. 1C) (15). When GroESL levels fall to 30% of the wild type (4 h depletion, Fig. 1D), cell lengths diverge from normal population lengths and the shallow constrictions are properly localized at midcell (Fig. 1C, Fig. S1).

10.1128/mBio.03564-20.1 FIG S1 Constrictions in early GroESL depletion are localized at midcell. Demograph showing width profiles of cell populations at 4 h of GroESL depletion. Cell populations are organized by cell length (*n* > 850, 2 pooled biological replicates). Download FIG S1, PDF file, 0.1 MB. Copyright © 2021 Schroeder et al. 2021 Schroeder et al. https://creativecommons.org/licenses/by/4.0/ This content is distributed under the terms of the Creative Commons Attribution 4.0 International license.

As protein folding is destabilized by temperature stress, we also investigated the heat sensitivity of the GroESL depletion strain, which at maximal xylose induction produces less GroESL (73%) than the wild type, and is unable to upregulate *groESL* transcription during stress conditions (Fig. 1B) (15). At the optimal growth temperature of 30°C, no difference in viability was observed between wild type C. crescentus and the GroESL depletion strain grown in the presence of xylose (Fig. 1E). However, a mild temperature increase to 36°C caused cultures of the GroESL depletion strain to filament and become inviable (Fig. 1C and D), emphasizing the importance of upregulating GroESL to provide chaperonin-mediated folding at elevated temperatures. Wild type C. crescentus also exhibits filamentation in response to diverse unfolding stresses (28), although a higher temperature of 40 to 42°C is required to elicit a similar response. Comparison of the insoluble, detergent-resistant protein fraction of wild type cultures with that of the GroESL depletion strain either during depletion or at elevated temperature (36°C) in the presence of xylose did not show that reduced GroESL had an effect on global protein solubility (Fig. 1F). Instead, only mild solubility changes in a small number of proteins were observed (Fig. 1F, arrows). Together, these results indicate that proteostasis and growth are generally maintained in the early stages of GroESL insufficiency. Therefore, the division defect is likely to result from the misfolding of one or more specific proteins linked to the cell cycle that depend on an interaction with GroESL for functionality.

### Chromosome replication and cell cycle transcription continue during GroESL insufficiency.

**FIG 2:**
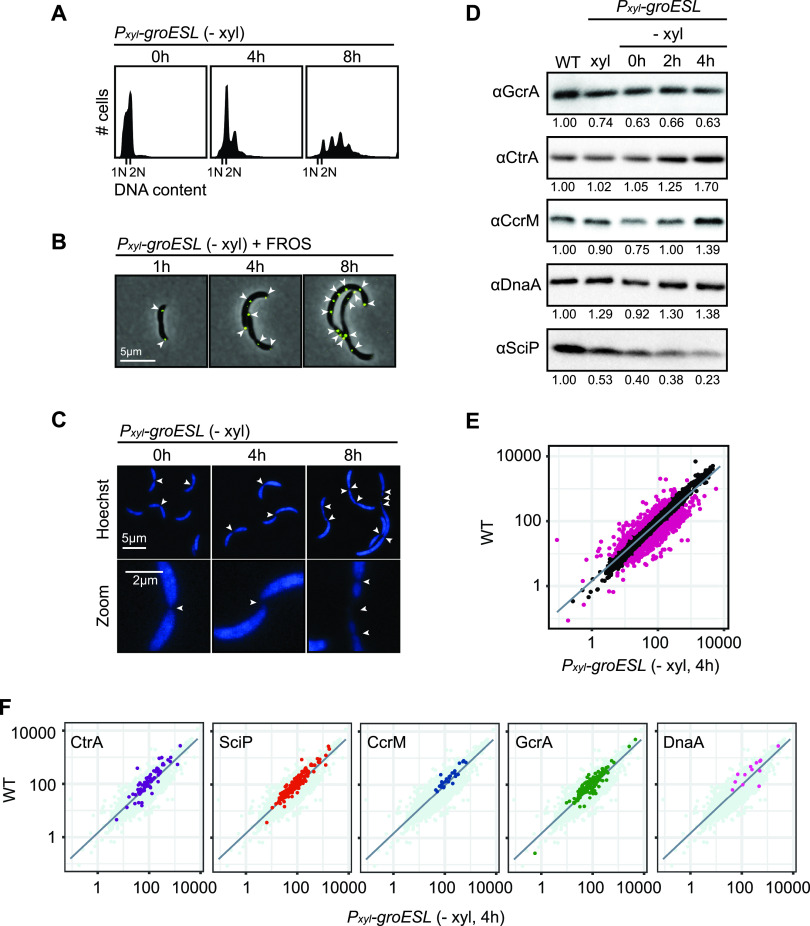
Chromosome replication and cell cycle transcription continue during GroESL insufficiency. (A) Flow cytometry profiles showing DNA content per cell at the indicated time points of GroESL depletion. (B) Microscopy of fluorescently labeled origins of replication at the indicated time points of GroESL depletion. A fluorescent reporter operator system (FROS) reporter construct bearing *ori*::(*tetO*)*n tetR-yfp* was used to mark origins of replication. Arrows indicate locations of origin of replication foci. Images show YFP-phase merge. (C) Microscopy of cells at the indicated time points during GroESL depletion, stained with Hoechst 33258 to visualize chromosomes. Arrows indicate gaps in staining associated with interchromosomal spaces. (D) Western blots of cell cycle regulators GcrA, CtrA, CcrM, DnaA, and SciP in wild type (WT) C. crescentus and the GroESL depletion strain (*P_xyl_-groESL*), grown either in non-depleting conditions (+ xyl) or for the indicated time periods in depleting conditions (− xyl). Quantification of band intensities represents an average of three biological replicates. (E) RNA-seq analysis showing normalized expression values for the C. crescentus transcriptome of wild type versus GroESL depletion at 4 h. Genes with a fold change of less than −2 or greater than 2 are represented by deep pink points. The line represents the smoothed conditional mean of the data. (F) Plots as in (E) with the genes belonging to the regulons of GcrA (34), CtrA (36), CcrM (32), DnaA (35), and SciP (33) highlighted.

C. crescentus filamentation can result from perturbations in DNA replication, chromosome segregation, the cell cycle transcriptional program, or inhibition of the cell division machinery. To identify which stage(s) of the cell cycle GroESL folding is required for, we assessed the consequences of GroESL depletion on each of these processes. Measuring DNA content by flow cytometry revealed that GroESL-depleting cultures accumulate additional chromosomes, even late in depletion (Fig. 2A), demonstrating that DNA replication continues when GroESL folding is insufficient. Consistent with this, multiple well-spaced origins of replication were distributed throughout the cytoplasm (Fig. 2B), and the chromosomes were spread throughout the entirety of the cell body (Fig. 2C). These data argue against a problem with chromosome segregation, which generally features chromosome-free spaces or mislocalized origins of replication (29).

The transcriptional circuit driving the cell cycle is poised to halt at the appearance of many stress inputs (28, 30, 31), therefore we assessed GroESL-depleted cells for the presence of the major cell cycle regulators CtrA, DnaA, CcrM, GcrA, and SciP, which drive the cell cycle-dependent transcriptional program in C. crescentus (32–36). CtrA, DnaA, GcrA, and CcrM remained at near wild type levels during the 4 h of GroESL depletion when the filamentation phenotype emerges, while SciP levels showed a reduction during this time frame (Fig. 2D). Like other components of the proteostasis network (12, 15), we found Lon levels to increase in GroESL-depleted cells (Fig. S2).

10.1128/mBio.03564-20.2 FIG S2 GroESL depletion induces changes in the proteostasis network and identity of insoluble proteins. (A) Western blot showing Lon abundance in wild type (WT) C. crescentus and the GroESL depletion strain (*P_xyl_-groESL*), grown either in non-depleting conditions (+ xyl) or for 0, 2, and 4 h in depleting conditions (− xyl). Quantification of band intensities represents an average of three biological replicates. (B) Prevalence of structural folds (SCOPe classification) in proteins of the detergent-resistant insoluble fraction of wild type C. crescentus cultures exposed to 45°C for one hour. The number of proteins indicates the absolute number of proteins identified with the indicated fold ID. The data set was obtained in a previous study (Schramm FD, Schroeder K, Alvelid J, Testa I, Jonas K. Mol Microbiol 111:1430–1448, 2019. https://doi.org/10.1111/mmi.14228). Download FIG S2, PDF file, 0.1 MB. Copyright © 2021 Schroeder et al. 2021 Schroeder et al. https://creativecommons.org/licenses/by/4.0/ This content is distributed under the terms of the Creative Commons Attribution 4.0 International license.

To more directly test if loss of GroESL-mediated folding affects cell cycle-regulated transcription in *Caulobacter*, we performed transcriptome sequencing (RNA-seq) comparing wild type C. crescentus transcription with that during early GroESL depletion (Fig. 2E, Table S1). This analysis showed that the transcriptional regulons controlled by all five cell cycle regulators, including SciP, remained largely unchanged when GroESL folding was reduced (Fig. 2F). Furthermore, we observed that levels of the SciP transcript were reduced 3.68-fold (Table S1). Together, these results suggest that DNA replication, chromosome segregation, and the cell cycle-dependent transcriptional program are not markedly affected by a reduction in available GroESL. Therefore, one or more proteins of the cell division apparatus may be specifically sensitive to the availability of GroESL, and mediate the filamentation phenotype of GroESL depletion.

10.1128/mBio.03564-20.7 TABLE S1 RNA-seq data Download Table S1, XLSX file, 0.1 MB. Copyright © 2021 Schroeder et al. 2021 Schroeder et al. https://creativecommons.org/licenses/by/4.0/ This content is distributed under the terms of the Creative Commons Attribution 4.0 International license.

### Loss of GroESL is associated with changes in the solubility of division and PG synthesis proteins.

**FIG 3:**
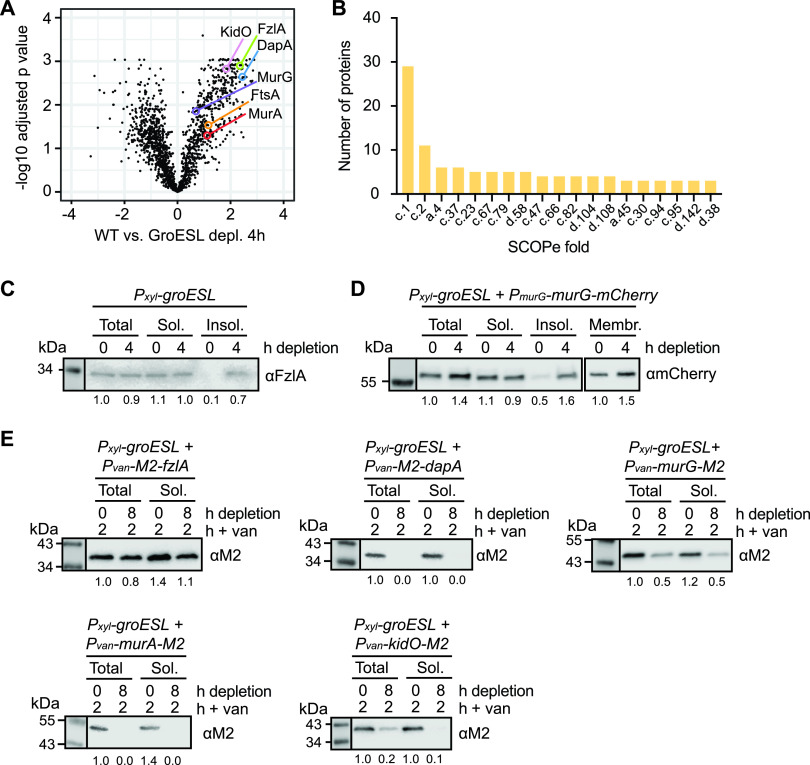
Loss of GroESL is associated with changes in division and cell wall synthesis protein solubility. (A) Changes in detergent-resistant insoluble fractions between wild type cultures and cultures depleted of GroESL for 4 h, identified by quantitative proteomics. The volcano plot shows significance (log adjusted *P* value, calculated using linear model analysis) versus log fold change for WT versus GroESL depletion at 4 h. Identified PG synthesis and cell division proteins are indicated. (B) Prevalence of structural folds (SCOPe classification) in proteins of the detergent-resistant insoluble fraction of cultures depleted of GroESL for 4 h. The number of proteins indicates the absolute number of proteins identified with the indicated fold ID. (C) Western blot of native FzlA abundance in cell lysate (total), soluble (sol.) and insoluble (insol.) cellular fractions of the GroESL depletion strain (*P_xyl_-groESL*). Samples were taken from cultures where GroESL was not depleted (0 h) as well as cultures where GroESL was depleted for 4 h. Band intensities are normalized to the amount of FzlA present in cell lysates at time 0 h. Quantification represents the average of at least three biological replicates. (D) Western blot of native MurG-mCherry abundance in cell lysate (total), soluble (sol.), insoluble (insol.), and membrane (membr.) cellular fractions of the GroESL depletion strain (*P_xyl_-groESL*). Samples were taken from cultures where GroESL was not depleted (0 h) as well as cultures where GroESL was depleted for 4 h. For total, soluble, and insoluble fractions, band intensities are normalized to the amount of MurG present in cell lysates at time 0 h, and for membrane fractions to the amount of MurG present in the membrane at time 0 h. Quantification represents an average of 3 biological replicates. (E) Solubility of *de novo*-synthesized M2-FzlA, M2-DapA, MurG-M2, MurA-M2, and KidO-M2 in cells depleted of GroESL (8 h). GroESL was depleted for 0 h or 6 h prior to induction of M2-FzlA, M2-DapA, MurG-M2, MurA-M2, and KidO-M2 for 2 h from the vanillate-inducible promoter (*P_van_*). Cultures were harvested and isolated into cell lysate (total) or soluble (sol.) fractions and immunoblotted. Band intensities are normalized to the amount of induced protein present after 2 h of induction in actively growing (+ xyl) cultures of the GroESL depletion strain (total fraction, 0 h depletion), and quantification represents an average of at least 3 biological replicates.

To identify candidate division-linked proteins whose folding is perturbed by reduced GroESL, we utilized a quantitative proteomics approach using isobaric tandem mass tag (TMT) mass spectrometry to identify proteins enriched in the insoluble, detergent-resistant fraction of cultures in early GroESL depletion (Fig. 3A). We identified 630 proteins whose presence in the insoluble fraction was significantly different between wild type and GroESL depletion, including 167 proteins with abundances increased at least 1.5-fold (*P* < 0.05) (Table S2). The best predictor of E. coli GroESL client proteins is a specific physicochemical signature (37), part of which is the presence of specific structural folds. Therefore, we analyzed the folds present in our identified population of enriched insoluble proteins. As in E. coli (18, 19, 38), we found the TIM beta/alpha barrel fold (c.1) to be overrepresented (Fig. 3B), with a 3.03-fold enrichment in proteins enriched in the GroESL-depleted insoluble fraction (12.393%) compared to the prevalence of this fold (4.086%) in the C. crescentus proteome. This enrichment of the c.1 fold is specific to GroESL depletion, as analysis of fold prevalence in the insoluble fraction of heat-stressed C. crescentus revealed other fold classes to be more prevalent under this condition (Fig. S2) (39). Among the proteins enriched in the insoluble fraction of GroESL-depleted cells, we identified five essential proteins that are linked to cell division and/or that function in the cell envelope: FzlA, FtsA, MurA, MurG, and DapA, as well as KidO, a nonessential oxidoreductase with a TIM beta/alpha barrel fold (Fig. 3A) (40, 41). FzlA, FtsA, and KidO are proteins that directly interact with FtsZ, the major structural component of the *Caulobacter* divisome (40–44), while MurA, MurG, and DapA are part of the PG biosynthesis pathway (45–48), which is critical for maintaining the cell envelope during normal growth and building the new poles during division.

10.1128/mBio.03564-20.8 TABLE S2 Proteomics data Download Table S2, XLSX file, 0.1 MB. Copyright © 2021 Schroeder et al. 2021 Schroeder et al. https://creativecommons.org/licenses/by/4.0/ This content is distributed under the terms of the Creative Commons Attribution 4.0 International license.

To validate the mass spectrometry results, we assessed the solubility of native FzlA and a MurG-mCherry fusion, integrated at the native chromosomal locus, when GroESL availability was reduced (Fig. 3C and D). This confirmed that these proteins are significantly enriched in the insoluble fraction when GroESL is depleted (Fig. 3C and D). Notably, a large proportion of both FzlA and MurG-mCherry was present in the soluble fractions. We reasoned this could be either due to the majority of the protein achieving a folded and soluble state before GroESL levels become limiting, or because most of the newly produced protein can correctly fold in the absence of GroESL. To discriminate between these possibilities, we assessed more directly how GroESL availability affects *de novo* production of the candidate proteins. For this, we tagged FzlA, KidO, DapA, MurA, and MurG with a small M2 tag, induced the expression of these fusion proteins for 2 h in either GroESL-depleted (6 h) or non-depleted conditions, and then quantified their abundance in the total and soluble protein fractions (Fig. 3E). We attempted to tag FtsA to include in this analysis, however as FtsA does not tolerate tags at either terminus, and tagging this protein has been demonstrated to alter its stability and function (49), we could not include it in our analysis. Our data show that similar amounts of M2-FzlA were present in the soluble and total protein fractions of GroESL-depleted and non-depleted cultures (Fig. 3E), indicating that FzlA can be produced and accumulate in a soluble state with reduced levels of GroESL. In contrast, the M2-DapA, MurG-M2, MurA-M2, and KidO-M2 fusions were only present at high levels when GroESL levels were sufficient for viability (Fig. 3E). In particular, *de novo*-synthesized DapA and MurA were not tolerated in cells lacking GroESL (Fig. 3E), therefore the accumulation of these proteins is strictly dependent on GroESL availability. We were however able to detect an enrichment in insoluble, native DapA and MurA in early GroESL depletion (Fig. 3A), indicating that synthesis of these proteins during insufficient GroESL folding results in production of insoluble protein, which is then degraded. Similar behavior is observed for several obligate E. coli GroESL substrates, including DapA (19, 20, 47). Our data suggest that DapA, MurA, MurG, and KidO could be GroESL clients in C. crescentus, as their accumulation depends on the presence of GroESL. As soluble FzlA was produced and accumulated in GroESL-depleted cultures, we conclude that this protein is unlikely to be an obligate GroESL client, but do not yet exclude a contribution to the cell division defect of GroESL-depleted cells.

### GroESL folding supports PG biosynthesis through MurG, MurA, and DapA.

**FIG 4:**
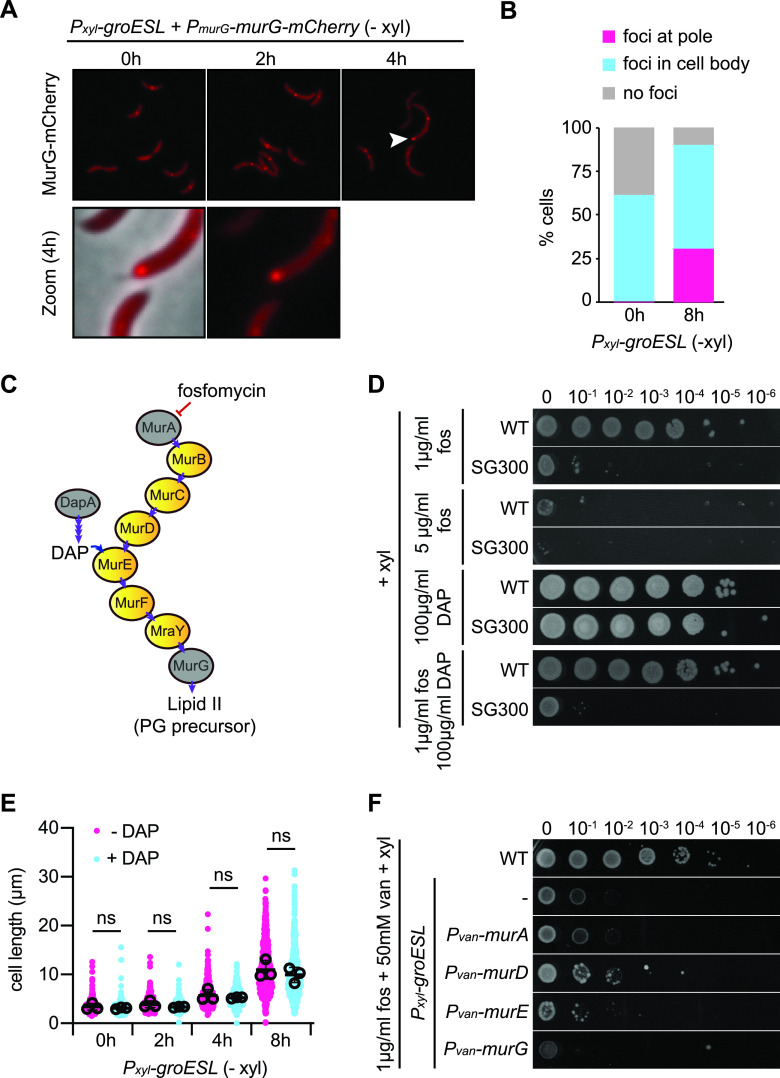
GroESL folding supports PG biosynthesis through MurG, MurA, and DapA. (A) Microscopy of MurG-mCherry localization during early GroESL depletion (0 to 4 h). Representative images are shown. The white arrow marks polar MurG-mCherry localization, magnified in lower panels. (B) Quantification of population location of MurG-mCherry localization patterns before (0 h) and after 8 h of GroESL depletion (*n* > 339, graph is the average of biological duplicates). Foci at pole (pink), cell contains at least one focus that is located in the extreme polar region; foci in cell body (blue), cell contains foci but not located at the pole; no foci (gray), cell contains only diffuse signal. (C) Schematic diagram of the PG biosynthetic pathway in C. crescentus. Proteins identified in Fig. 3A are highlighted in gray. Important metabolites (DAP, Lipid II) are indicated where they appear in the pathway, as well as where fosfomycin acts to inhibit MurA. Purple arrows indicate enzymatic reactions. (D) Spot assay of wild type and the GroESL depletion strain (*P_xyl_-groESL*) in the presence of fosfomycin (fos) and/or DAP. Xylose was included in all agar plates. Images are representative of 3 biological replicates. (E) Quantification of population cell lengths (*n* = 200 for each of three biological replicates) determined by phase-contrast microscopy of the GroESL depletion strain (*P_xyl_-groESL*) during depletion (− xyl) in the presence or absence of 100 μg/ml DAP (ns, no significant difference). (F) Spot assay of wild type and derivatives of the GroESL depletion strain (*P_xyl_-groESL*) harboring chromosomally-encoded, inducible genes encoding the PG biosynthetic pathway proteins MurA-M2, MurD-M2, MurE-M2, or MurG-M2 when treated with fosfomycin (fos). Xylose was included in all agar plates to support GroESL expression, and vanillate was included to induce the expression of PG biosynthesis proteins. The GroESL depletion strain (*P_xyl_-groESL*) without integrated plasmids is included as a control (−). Images are representative of 3 biological replicates.

DapA, MurA, and MurG are all part of the PG biosynthetic pathway, which functions to build and maintain the outer structure of growing cells, including the new cell poles during division (16). We first focused on this group of proteins and investigated the relationship between PG biosynthesis and GroESL folding. First, we sought additional support for our data that the divisome-associated protein MurG may require an interaction with GroESL to reach a functional state and determined the localization of MurG-mCherry during GroESL depletion. We found that MurG-mCherry formed multiple foci along the length of the cell (Fig. 4A), which may be indicative of aggregation (39), or alternatively, as MurG function is associated with FtsZ and the Z-ring (26, 45, 48), its association with partially assembled or partially functional divisome components. Importantly, we observed that in a subpopulation of cells (30%), MurG-mCherry formed polar foci (Fig. 4A and B). These polar MurG-mCherry foci did not occur in non-depleting conditions, suggesting they are caused by reduced GroESL availability. As condensation of *Caulobacter* FtsZ, and therefore the divisome, is inhibited at the poles (50) and furthermore as the *Caulobacter* poles are stable regions where new PG is not inserted (45), this observation is consistent with MurG-mCherry clustering in a nonfunctional state.

PG precursors are built in the cytoplasm through the sequential action of a series of enzymes, including MurA and MurG, and metabolites for the pathway are supplied by DapA (Fig. 4C). The first committed step of PG biosynthesis requires the activity of MurA, which is targeted by the antibiotic fosfomycin (51, 52). To assess the stability of PG biosynthesis when GroESL folding is reduced, we determined the sensitivity of the GroESL depletion strain, grown in non-depleting conditions, to fosfomycin. Interestingly, the GroESL depletion strain demonstrated hypersensitivity toward this antibiotic (Fig. 4D), and additionally was hypersensitive to the cell wall-targeting antibiotic vancomycin (to which C. crescentus is sensitive) (53), though showed no increased sensitivity to the cephalosporin antibiotic cefixime, suggesting that penicillin-binding proteins (PBPs) are not involved in the phenotype (Fig. S3). Together, these data indicate that PG biosynthesis is highly sensitive to changes in GroESL availability. In E. coli, DapA is an obligate GroESL substrate that catalyzes the formation of 4-hydroxy-tetrahydrodipicolinate, a precursor of meso-diaminopimelate (DAP) that is required for normal PG synthesis (47). Addition of DAP to the growth medium prevents lysis due to DapA degradation in GroESL-depleted E. coli (47); therefore, we supplemented fosfomycin-treated C. crescentus cultures with DAP in an attempt to bypass the essential function of this protein (Fig. 4D). DAP supplementation was unable to restore fosfomycin resistance (Fig. 4D), therefore we also tested the effect of DAP on GroESL-depleting cultures in the absence of the antibiotic. C. crescentus supplemented with DAP still became filamentous during GroESL depletion, with no reduction in cell length indicating this metabolite is unable to improve the phenotype of insufficient GroESL in C. crescentus (Fig. 4E, Fig. S4). These data are consistent with the finding that DapA is not the only protein of the PG biosynthetic pathway that has solubility changes when GroESL levels become limiting, but that the proteins MurA and MurG may also require GroESL-mediated folding.

10.1128/mBio.03564-20.3 FIG S3 Insufficient GroESL causes hypersensitivity to certain cell wall-targeting antibiotics. (A) Spot assay of wild type and GroESL depletion strain (*P_xyl_-groESL*) in the presence of vancomycin (vanc). Xylose was included in all agar plates. Images are representative of 3 biological replicates. (B) Spot assay of wild type and GroESL depletion strain (*P_xyl_-groESL*) in the presence of cefixime (cef). Xylose was included in all agar plates. Images are representative of 3 biological replicates. (C) Spot assay of wild type and derivative of the GroESL depletion strain (*P_xyl_-groESL*) harboring a chromosomally encoded, inducible M2-MreB when treated with fosfomycin (fos). Xylose was included in all agar plates to support GroESL expression, and vanillate was included to induce the expression of MreB. The GroESL depletion strain (*P_xyl_-groESL*) without integrated plasmids was included as a control (−). Images are representative of 3 biological replicates. (D) Spot assay of wild type and GroESL depletion strain (*P_xyl_-groESL*) in the presence of the MreB inhibitor A22. Xylose was included in all agar plates. Images are representative of 3 biological replicates. Download FIG S3, PDF file, 0.2 MB. Copyright © 2021 Schroeder et al. 2021 Schroeder et al. https://creativecommons.org/licenses/by/4.0/ This content is distributed under the terms of the Creative Commons Attribution 4.0 International license.

10.1128/mBio.03564-20.4 FIG S4 Growth curve during DAP supplementation in GroESL depletion. Growth curve assessing biosynthetic capacity of wild type and GroESL depletion strains in the presence and absence of 100 μg/ml DAP. Cultures were prepared at an OD of 0.1 and washed of xylose prior to adding to the plate containing the appropriate additives where necessary. Download FIG S4, PDF file, 0.1 MB. Copyright © 2021 Schroeder et al. 2021 Schroeder et al. https://creativecommons.org/licenses/by/4.0/ This content is distributed under the terms of the Creative Commons Attribution 4.0 International license.

To address the possible interaction of other PG biosynthetic pathway enzymes with GroESL, we attempted to increase the activity of these proteins by increasing their gene copy number and therefore expression levels, a method that has been used to investigate the contribution of specific clients to the phenotype of GroESL depletion in E. coli (14). Increased expression of these proteins did not improve fosfomycin sensitivity (Fig. 4F), suggesting again that the PG biosynthesis pathway has multiple points of interaction with GroESL. We additionally tested the involvement of MreB, which organizes PG insertion in *Caulobacter* and is classified as a class II, nonobligate substrate of E. coli GroESL (19, 48), and also as a client of DnaK (54). However, increased expression of the actin homologue MreB did not rescue PG hypersensitivity, and the GroESL depletion strain was not more sensitive to the MreB inhibitor A22 than was wild type at the concentrations tested (Fig. S3). Together, our results indicate that GroESL supports the folding and solubility of several proteins of the PG biosynthesis pathway, including MurG, MurA, and DapA. Decreased GroESL folding capacity results in reduced functionality of this pathway, consequently increasing fosfomycin sensitivity.

### Constriction stalls and the Z-ring is mislocalized shortly after GroESL levels begin to decline.

**FIG 5:**
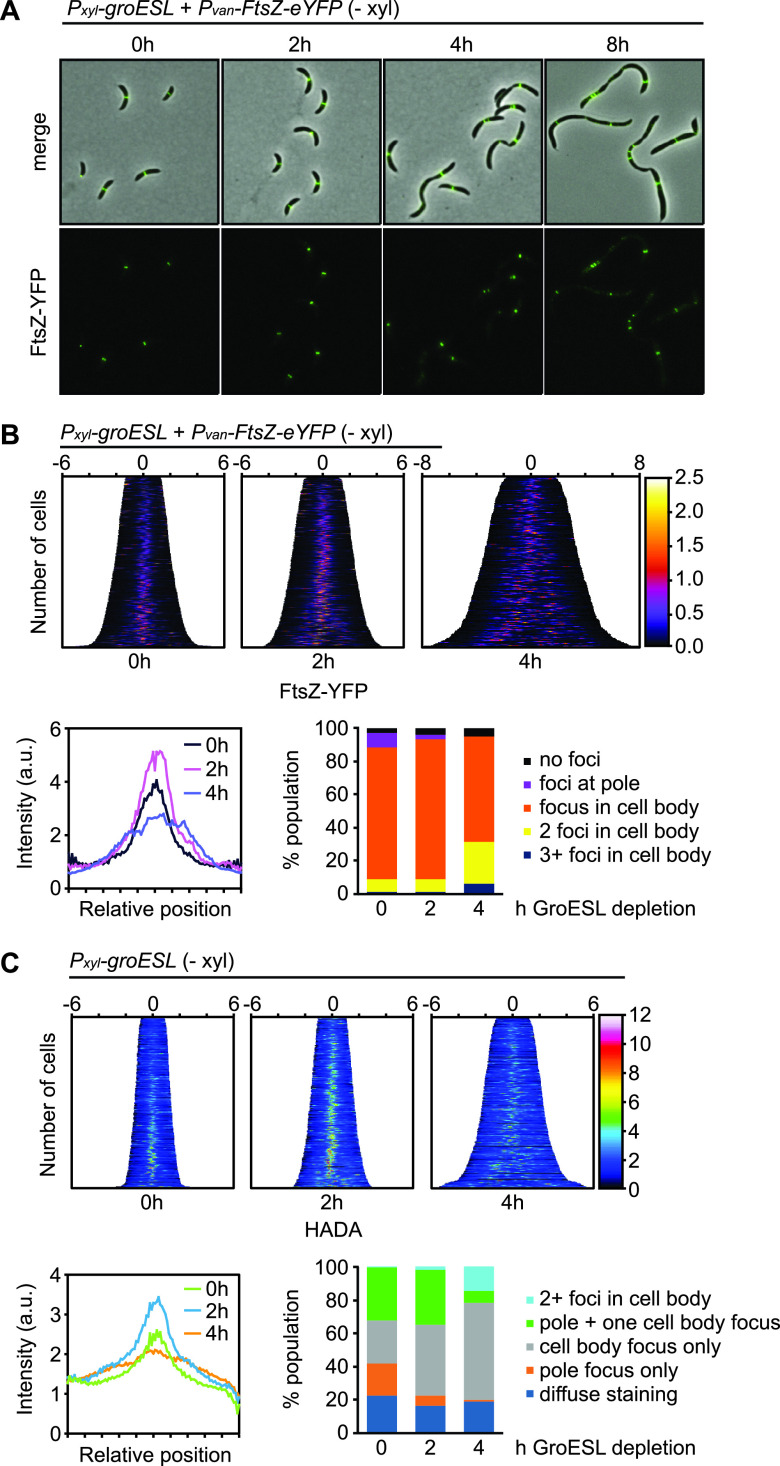
Constriction stalls and the Z-ring is mislocalized shortly after GroESL levels decline. (A) Microscopy of FtsZ-eYFP localization in the GroESL depletion strain during depletion (− xyl). FtsZ-eYFP was expressed from the vanillate-inducible promoter (*P_van_-ftsZ-eYFP*) for 2 h prior to imaging at each time point of GroESL depletion. Representative micrographs are shown. (B) Top: demographs showing fluorescent signal profiles of FtsZ-eYFP in early GroESL depletion (0 to 4 h), organized by cell length (*n* > 308 each population). Fluorescent profiles are organized by cell length. Bottom, left: average FtsZ-eYFP fluorescence intensity across normalized cell length for populations shown in the demographs. Bottom, right: population fractions with different FtsZ-eYFP foci number and position (*n* > 230 for each of three biological replicates). (C) Top: demographs of population fluorescence intensity profiles of HADA stain (*n* > 334 each population). Cultures were depleted of GroESL for the indicated time periods and exposed to a short pulse (2 min) of HADA prior to fixation and imaging. Population intensity profiles are organized by cell length. Bottom, left: average HADA fluorescence intensity across normalized cell length for populations shown in the demographs. Bottom, right: population fractions with different HADA foci number and position (*n* > 150 for each of three biological replicates).

In addition to proteins required for PG biosynthesis, we identified FzlA, FtsA, and KidO as being enriched in the insoluble fraction of GroESL-depleted cells (Fig. 3A). All three of these proteins interact with FtsZ, and FzlA and FtsA provide essential regulation of FtsZ polymer formation and consequently its function in coordinating cell division (42, 44, 53, 55). Therefore, we determined the effects of GroESL depletion on the formation and function of the Z-ring. We first assessed the condensation of FtsZ during GroESL depletion using a merodiploid FtsZ-eYFP fusion reporter (Fig. 5A) (50). We found that before significant GroESL-mediated cell length changes occur during GroESL depletion (2 h time point), the Z-ring was present at midcell in a larger proportion of the population than in actively dividing cells (Fig. 5B). By 4 h depletion, multiple FtsZ foci were present in disorganized locations along the cell length, with no obvious bias in positioning other than that FtsZ foci were excluded from the poles (Fig. 5A and B). Our data are consistent with the persistence of multiple Z-rings that has previously been observed by immunofluorescent staining in late phase (10 h) GroESL depletion (15). Therefore, when GroESL levels are reduced, FtsZ polymerizes and condenses but constriction stalls before division is complete, with many of the Z-rings assembled within 2 h of GroESL depletion failing to achieve division. The ability to divide is lost asynchronously, as division is observed to occur in some cells later in depletion, suggesting that the remaining chaperonin may occasionally provide enough folding of the required division protein(s) (Movie S1). Collectively, these results indicate that stalling in constriction associated with changes in Z-ring localization immediately precede the cell length changes observed during early GroESL depletion, suggesting that misfolding of an FtsZ-interacting protein could be the primary driver of the cell division defect.

10.1128/mBio.03564-20.10 MOVIE S1 Dynamics of FtsZ condensation during GroESL depletion. Download Movie S1, AVI file, 2.0 MB. Copyright © 2021 Schroeder et al. 2021 Schroeder et al. https://creativecommons.org/licenses/by/4.0/ This content is distributed under the terms of the Creative Commons Attribution 4.0 International license.

To investigate altered Z-ring localization without the influence of the inducible fluorescent fusion construct, we further assessed GroESL depletion using a fluorescent-d-amino acid (HADA) (Fig. 5C), which marks the active PG incorporation at midcell that is coordinated by FtsZ, as well as that coordinated by the elongasome (56) and during stalk elaboration (57). In agreement with the FtsZ fluorescent fusion, we observed that a higher proportion of the population exhibited a bright midcell focus of FtsZ-localized PG incorporation at 2 h GroESL depletion, in contrast to non-depleted cultures (Fig. 5C). Additionally, foci became disorganized and were found along the cell length at later time points (Fig. 5C, Fig. S5), indicating that the assembled Z-rings continue to coordinate PG insertion while being unable to complete cell division. Furthermore, we observed that foci of PG insertion at the pole, reflective of stalk elaboration in actively dividing populations, was present in a very small proportion of the population during GroESL depletion, consistent with our hypothesis that some of the MurG present in GroESL-depleted cells is clustering in a nonfunctional, insoluble state.

10.1128/mBio.03564-20.5 FIG S5 HADA staining reveals disorganized PG incorporation. Representative images of C. crescentus showing different HADA staining patterns used for quantification in Fig. 5C and Fig. 7A. Polar foci are consistent with PG synthesis during stalk elaboration. A, pole focus only; B, diffuse staining; C, pole and one cell body focus; D, cell body focus only; E, 2+ foci in cell body; F and G, images of disorganized HADA staining patterns observed at 8 h GroESL depletion. Download FIG S5, PDF file, 0.3 MB. Copyright © 2021 Schroeder et al. 2021 Schroeder et al. https://creativecommons.org/licenses/by/4.0/ This content is distributed under the terms of the Creative Commons Attribution 4.0 International license.

### GroESL-mediated changes in constriction can be compensated by FtsA and FzlA.

**FIG 6:**
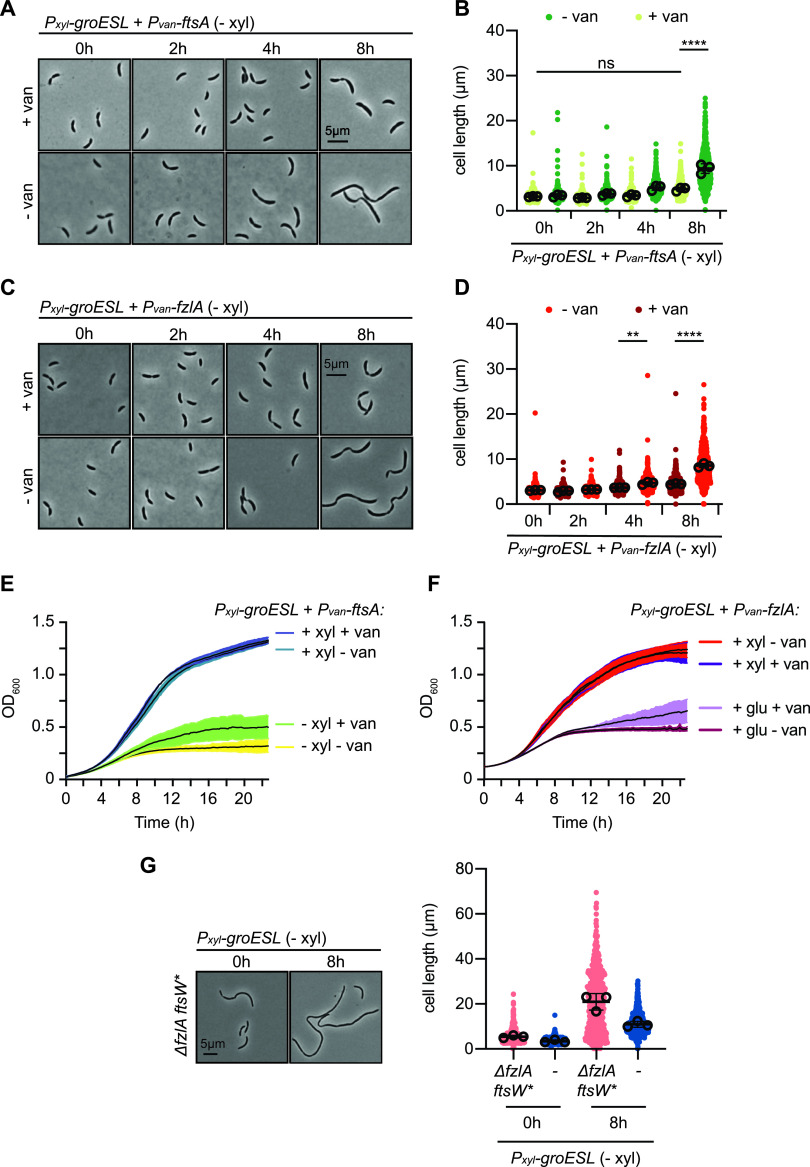
GroESL folding regulates constriction through FtsA and FzlA. (A) Microscopy of GroESL depletion with induced expression of *ftsA* from a second chromosomal locus. Vanillate-dependent *ftsA* expression was induced at the onset of GroESL depletion (0 h). Microscopy images of isogenic cultures during GroESL depletion, grown without the addition of vanillate (− van) are shown for comparison. (B) Quantification of population cell lengths from (A) (*n* = 200 for each of three biological replicates). Isogenic cultures were grown with (+ van) or without (− van) the addition of vanillate and population cell lengths were quantified (ns, no significant difference; ****, *P* < 0.0001). (C) Microscopy of GroESL depletion with induced expression of *fzlA* from a second chromosomal locus. Vanillate-dependent *fzlA* expression was induced at the onset of GroESL depletion (0 h). Microscopy images of isogenic cultures during GroESL depletion, grown without the addition of vanillate (− van) are shown for comparison. (D) Quantification of population cell lengths from (C) (*n* = 200 for each of three biological replicates). Isogenic cultures were grown with (+ van) or without (− van) the addition of vanillate and population cell lengths were quantified (ns, no significant difference; **, *P* < 0.01; ****, *P* < 0.0001). (E) Growth curve assessing biosynthetic capacity of the GroESL depletion strain (*P_xyl_-groESL*) producing additional FtsA. Isogenic cultures were grown in the presence or absence of xylose (+/− xyl) and the presence or absence of vanillate (+/− van) to determine growth effects. (F) Growth curve assessing biosynthetic capacity of the GroESL depletion strain (*P_xyl_-groESL*) producing additional FzlA. Isogenic cultures were grown in the presence or absence of xylose (+/− xyl) and the presence or absence of vanillate (+/− van) to determine growth effects. (G) Left: Microscopy of strains lacking FzlA (ΔfzlAftsW*) before and 8 h after GroESL depletion. Right: Quantification of population cell lengths (*n* > 140 for each of three biological replicates) compared with population cell lengths of the parent GroESL depletion strain (shown in blue).

Because both FzlA and FtsA are critical for regulating FtsZ dynamics (42, 44, 55), we hypothesized that incorrect or insufficient folding of either, or both, of these proteins may lead to the observed changes in FtsZ behavior at the early stages of GroESL depletion. We therefore sought to evaluate if increased production of FzlA or FtsA could reduce or delay the effects of insufficient GroESL folding and alleviate the GroESL depletion phenotype. Strikingly, when either FzlA or FtsA were produced from an additional chromosomal locus we observed a significant delay in the development of filamentation during GroESL depletion (Fig. 6A to D, Fig. S6). Cell lengths were similar during GroESL depletion for cultures expressing either extra FtsA or FzlA, and in both cases were significantly shorter (Fig. 6A to D), suggesting additional division events had occurred. It is important to note that FtsA expression levels in this genetic context did not lead to the filamentation phenotypes observed with strong overexpression of FtsA in wild type C. crescentus (Fig. S6) (58), and that strong overexpression of FzlA does not lead to cell length changes, even in a wild type context (44). Growth analysis revealed that production of extra FtsA or FzlA was able to improve growth capacity (Fig. 6E and F), thus excluding the possibility that the shorter cells observed during GroESL depletion in the presence of additional FtsA or FzlA were due to growth arrest or a decrease in growth rate.

10.1128/mBio.03564-20.6 FIG S6 Phenotype of *ftsA*, *fzlA*, and *ftsE* expression from a second chromosomal locus. (A) Western blot showing FzlA abundance in the GroESL depletion strain (*P_xyl_-groESL*), grown in non-depleting conditions (+ xyl) after 3 h of induction (+ van). (B) Microscopy of induced expression of *ftsA* from a second chromosomal locus in wild type and GroESL depletion strain (+ xyl). Microscopy images are shown from exponentially growing cultures prior to *ftsA* induction (0 h), and after vanillate-dependent *ftsA* expression was induced and maintained in exponentially growing cultures for 6 h. (C) Quantification of population cell lengths of strains expressing FtsA, FzlA, or FtsE from an additional chromosomal locus before and eight hours after GroESL depletion (− xyl). Populations of *n* = 200 for each of three biological replicates were measured (ns, no significant difference; *** *P* < 0.001). (D, top) Microscopy of Δ*ftsE* strain before and eight hours after GroESL depletion. (D, bottom) Quantification of population cell lengths (*n* > 130 for each of three biological replicates), compared with population cell lengths of the parent GroESL depletion strain (shown in blue). Download FIG S6, PDF file, 0.3 MB. Copyright © 2021 Schroeder et al. 2021 Schroeder et al. https://creativecommons.org/licenses/by/4.0/ This content is distributed under the terms of the Creative Commons Attribution 4.0 International license.

To attempt to distinguish between the contributions of FtsA and FzlA to the GroESL depletion division defect, we evaluated the effects of GroESL depletion on Z-ring function in the absence of FzlA. For this, we made use of a previously established Δ*fzlA* suppressor strain in which a point mutation in FtsW (A246T) compensates for loss of the essential function of FzlA (53), thus permitting cell division in its absence. Depletion of GroESL in the Δ*fzlA* suppressor strain exacerbated the filamentation phenotype (Fig. 6G), and the development of irregularly spaced constrictions was still observed (Fig. 6G). These data indicate that aberrant interaction of misfolded FzlA with the divisome is not responsible for the GroESL depletion cell division defect, and additionally suggests that another factor is involved. As FtsA depletion results in filamentous cells with shallow, irregularly spaced constrictions that resemble the phenotype of GroESL depletion (59), our data suggest that FtsA could be the primary driver of the GroESL depletion division block.

Finally, to determine if increased expression of other Z-ring-interacting proteins might be able to improve the division defect of insufficient GroESL, we introduced a second chromosomal copy of the nonessential ABC transporter and FtsZ-interacting protein FtsE (60), chosen also due to its involvement in the GroESL depletion phenotype of E. coli (19, 21). Increasing FtsE levels did not result in the same reduction in cell length as increasing FzlA or FtsA (Fig. S6), and the GroESL depletion phenotype developed similarly in a C. crescentus strain lacking *ftsE* (Fig. S6). Together, these results indicate that a function common to FtsA and FzlA, and not to all FtsZ-binding proteins, is disrupted by insufficient GroESL folding.

### Increased FtsA compensates for GroESL depletion in optimal and proteotoxic stress conditions.

**FIG 7:**
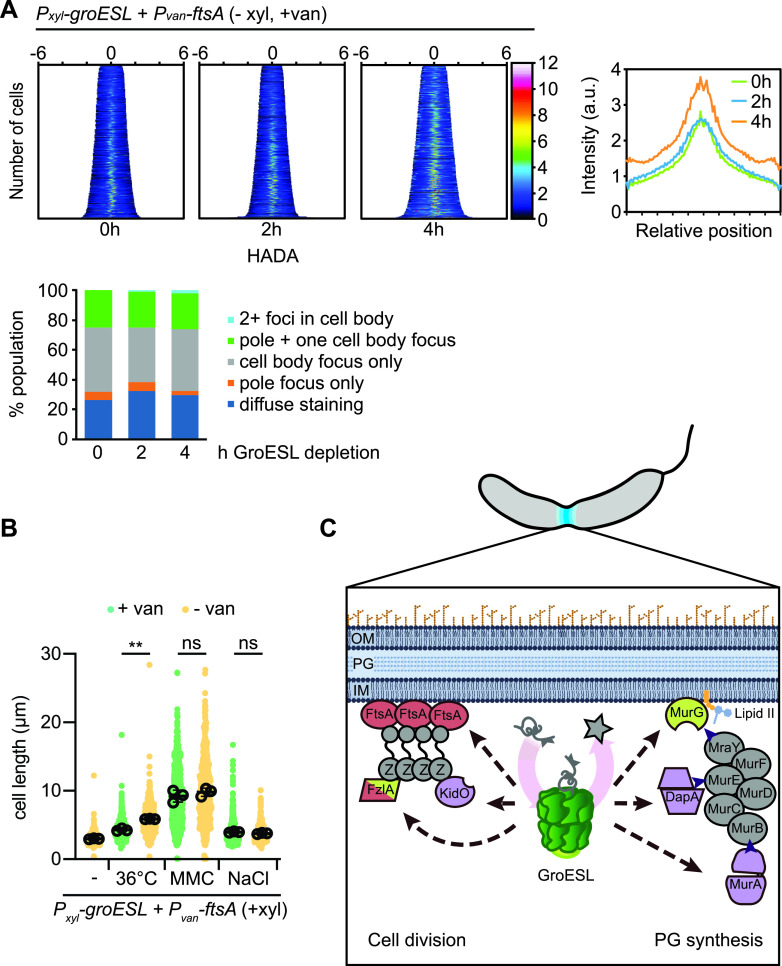
Increased FtsA compensates for GroESL depletion in optimal and stress conditions. (A) Top, left: Population fluorescence intensity profiles of HADA staining for populations of GroESL-depleted cultures producing extra FtsA (*n* > 502, each population). Cultures were depleted of GroESL for the indicated time periods with vanillate-dependent expression of FtsA induced at the onset of depletion (0 h). At the indicated time points cultures were exposed to a short pulse (2 min) of HADA prior to fixation and imaging. Population intensity profiles are organized by cell length. Top, right: Average HADA fluorescence intensity across normalized cell length for populations shown in demographs. Bottom, left: Population fractions with different HADA foci number and position (*n* > 200 for each of three biological replicates). (B) Quantification of population cell lengths of cultures exposed to stress while producing additional FtsA. Isogenic cultures in non-depleting conditions (+ xyl) were grown with (+ van) or without (− van) vanillate during exposure to 36°C, 3 μg/ml mitomycin C (MMC), or 85 mM NaCl. Population cell lengths (*n* = 200 for each of three biological replicates) were quantified (ns, no significant difference; **, *P* < 0.01). (C) Schematic showing GroESL supports the function of several division proteins and peptidoglycan biosynthetic enzymes during C. crescentus division. The chaperonin GroESL is a foldase that assists in moving client proteins from folding intermediates to native folded proteins (star). GroESL folding supports the solubility of FtsA, FzlA, and KidO in the divisome, and MurG, DapA, and MurA in the PG biosynthetic pathway (metabolite flow from candidate GroESL client proteins is indicated by arrowheads). Proteins in red (FtsA, FzlA) are able to temporarily rescue the GroESL depletion filamentation phenotype if provided in excess. FtsA interacts with the C-terminal conserved region of FtsZ (Z) to anchor FtsZ filaments to the membrane and regulate its dynamics during division. FzlA interacts with the GTPase domain of FtsZ to introduce FtsZ polymer curvature and regulate constriction. Proteins in purple (DapA, MurA, KidO) are degraded if synthesized in the absence of sufficient GroESL folding. Proteins in green exhibit altered localization. Proteins in gray do not decrease solubility during GroESL depletion. Dashed lines represent interactions that may proceed through an as of yet unidentified intermediate. OM, outer membrane; PG, peptidoglycan; IM, inner membrane. Membrane and PG images created with Biorender (Biorender.com).

As FzlA and FtsA both act on FtsZ and are both able to improve the GroESL depletion cell division block, we continued to assess the effects of increased abundance of these proteins using FtsA. To evaluate the impact of providing extra FtsA on placement of FtsZ-localized PG metabolism during early GroESL depletion (Fig. 5C), we again used HADA staining. During the FtsA-mediated delay in constriction stalling, the proportion of newly divided cells without a midcell focus of PG incorporation was maintained for an additional 2 h, or at least one additional population doubling (Fig. 7A versus Fig. 5C), during which growth rate was maintained. These data are consistent with increased FtsA production compensating for a reduction in the ability to efficiently fold FtsA, and improving Z-ring positioning during GroESL depletion.

Finally, we assessed the ability of additional FtsA to delay filamentation in other conditions, including growth at 36°C, when GroESL is also insufficient. Expression of additional FtsA was unable to reduce cell length changes resulting from DNA damage, nor from salt stress (Fig. 7B), where CtrA is inactivated and unable to stimulate FtsA expression (28). However, expression of additional FtsA resulted in a cell length reduction in cultures exposed to heat stress (Fig. 7B), suggesting that sufficient levels of GroESL are also required to promote division via an interaction with FtsA during unfolding stress. Collectively, these experiments illustrate that GroESL is necessary to support normal Z-ring function during division, at least in part via the FtsZ-anchoring protein FtsA, which is sensitive to the folding capacity of the chaperonin. Furthermore, our data indicate that C. crescentus also tunes cell division to chaperone availability via FtsZ-interacting proteins, in part through an actin protein-chaperonin interaction.

## DISCUSSION

Chaperonins are highly conserved folding machines that provide essential protein folding across all kingdoms of life. Critical functions of chaperonins range from helping bacteria to build peptidoglycan (47) to supporting chloroplast and mitochondrial function in eukaryotes (61, 62), and information from prokaryotic systems has helped to inform exploration of human chaperonins (63). In this present work we expand on how chaperonin function is integrated into bacterial physiology by exploring GroESL function in the alphaproteobacterium Caulobacter crescentus. We find that the integration of GroESL into the processes of cell division and synthesis of the cell envelope is conserved among different groups of bacteria; however, this integration occurs via distinct points of interaction (Fig. 7C). In C. crescentus, GroESL folding is required to support PG biosynthesis via MurG, MurA, and DapA, but is most critically required to support cell division through interactions with FtsA and FzlA. By linking a chaperonin to these processes, stress-responsive protein folding capacity is intimately connected to both cell envelope synthesis and cell division in *Caulobacter*.

Our study has shown that chaperonin folding is indispensable for PG synthesis in C. crescentus, and has identified several new interactions between PG biosynthetic proteins and GroESL. This is the first description of MurA being linked to GroESL folding, though an interaction with DnaKJE has previously been established (54). Our data suggest that MurG solubility and localization may also respond to GroESL-mediated folding (Fig. 4A). MurG has been shown to act as a scaffold for PG biosynthesis in Bordetella pertussis and Thermotoga maritima (64, 65) and could provide a similar function in C. crescentus, though it remains unclear if the changes we observe for MurG are due to absence of a direct interaction with GroESL, or perhaps loss of an upstream signal required for PG biosynthetic subcomplex assembly. Depletion of PG precursors, which may occur through a reduction in pathway protein function, results in filamentation to conserve limited resources and prevent overinvestment in the intensive process of building new cell poles (51). During periods of proteotoxic stress and high refolding demand, titration of the chaperonin away from synthetic processes could provide a way to postpone cell division and focus on survival. Interaction of both DnaKJE and GroESL with unfolded proteins is known to regulate the heat shock response to this end (5), and DnaKJE availability during stress is integrated into the cell cycle as an indirect regulator of DNA replication initiation (31). It remains to be discovered how PG synthetic protein folding and abundance are prioritized during stress. Newly discovered accessory factors, such as the holdase CnoX (66), may hold the key to how client proteins are presented to GroESL, and which processes are protected during high unfolding demand. Discerning these interactions will be important to understanding how organisms balance growth and division when surviving stress.

Our study has identified the bacterial actin homologue FtsA and the constriction regulator FzlA, both interactors of the tubulin homologue FtsZ, as proteins that are particularly sensitive to GroESL availability. FtsA and FzlA were among the proteins that showed increased insolubility in the absence of GroESL (Fig. 3A) and, although the enrichment of FtsA in the insoluble fraction was mild, producing either extra FtsA or FzlA was able to delay the development of filamentation as chaperonin levels declined (Fig. 6A to D). Work in E. coli has shown that increasing the expression of other GroESL client proteins (or those that feed into the client protein function) can also temporarily compensate for reduced levels of GroESL during depletion (14, 21) by providing a reserve pool of folded client protein to draw on. Our data linking FtsA function with GroESL is particularly striking, as a major role of the eukaryotic chaperonin TRiC is to perform folding of eukaryotic actin (67, 68), yet bacterial FtsA has not been identified previously as an interactor with GroESL or DnaKJE (19, 54). Therefore, our findings raise questions on the conservation of the relationship between actin proteins and chaperonins. As FtsA is also a highly conserved and crucial protein in diverse bacteria, it will be important to determine the relationship between actin homologues and GroESL in other organisms, including clarifying this relationship in E. coli.

We additionally observed that increased abundance of the constriction regulator FzlA was able to compensate for insufficient GroESL levels. Loss of FzlA function in modulating constriction rate is associated specifically with increased sensitivity to PBP-targeting antibiotics but not vancomycin or fosfomycin (53), the inverse phenotype to that observed in the GroESL depletion strain (Fig. S3). Furthermore, as a large proportion of soluble FzlA can be synthesized when GroESL levels are reduced (Fig. 3C and E), it remains unclear how increasing FzlA levels is able to compensate for a reduction in GroESL-mediated folding. FzlA binds the GTPase domain of FtsZ, and FtsA the C-terminal conserved region (43, 69), and it remains to be determined if increased FzlA could introduce alterations in FtsZ polymer structure that may compensate for the effects of reduced FtsA availability, or if another as of yet unidentified interaction exists between FzlA and FtsA.

Finally, our work indicates that the E. coli obligate GroESL client protein FtsE is not the primary driver of the GroESL depletion division defect in C. crescentus (19, 21), suggesting that different organisms have evolved separate links between GroESL and cell division. As GroESL is thought to support evolutionary plasticity in metabolic enzymes (70), it is an open question whether the chaperonin might permit similar flexibility in cell division proteins, a question with consequences for resistance to current and future antimicrobials that target the cell envelope and cell division.

## MATERIALS AND METHODS

### Strains and plasmids.

The strains and plasmids used in this study are listed in Table S3 in the supplemental material.

10.1128/mBio.03564-20.9 TABLE S3 Strain and plasmid list Download Table S3, XLSX file, 0.01 MB. Copyright © 2021 Schroeder et al. 2021 Schroeder et al. https://creativecommons.org/licenses/by/4.0/ This content is distributed under the terms of the Creative Commons Attribution 4.0 International license.

### Bacterial growth conditions.

All C. crescentus strains were routinely cultured at 30°C, unless otherwise indicated, in liquid peptone yeast extract (PYE) medium with shaking at 200 rpm. If necessary, the following medium supplements were added to the following final concentrations: 0.3% xylose, 0.2% glucose, 25 μg/ml spectinomycin, 5 μg/ml kanamycin, 0.625 μg/ml gentamicin, 500 mM vanillate. Cultures were regularly diluted to keep them in mid-log phase. In GroESL depletion experiments, cells were washed three times with PYE free of medium supplements by centrifugation (6,000 × *g*, 4 min) before resuspension in medium lacking xylose inducer. Growth on solid PYE medium was performed in the presence of the following supplement concentrations: 0.3% xylose, 0.2% glucose, 500 mM vanillate, 5 μg/ml gentamicin, 25 μg/ml kanamycin, 400 μg/ml spectinomycin. Transductions were performed using ϕCr30 as described previously (71). E. coli was grown for cloning purposes in LB supplemented with antibiotics as necessary at 37°C.

### Spot assays.

Spot assays were performed with cultures maintained in log phase for 3 h and diluted to an optical density at 600 nm (OD_600_) of ∼0.2. Tenfold serial dilutions of this culture were prepared and 2 μl of each dilution was spotted and dried onto a fresh agar plate.

### Growth curves.

For growth curve experiments, cultures maintained in log phase for 3 h were diluted to an OD_600_ of ∼0.05 and 200 μl of diluted culture was added to 96-well plates. Measurement was performed every 10 min at 30°C with culture aeration in a Tecan Spark for 24 h. Three biological replicates were performed for all growth curve measurements, with three technical replicates for each sample.

### Western blotting.

For total cell protein detection, cell pellets were harvested by centrifugation and resuspended in Laemmli buffer normalized to OD_600_ measurement, followed by heating at 70°C for 10 min. For fractionated samples, dilution in Laemmli buffer was normalized according to lysate protein concentration, with insoluble fractions concentrated 20× to account for the lower relative abundance of this fraction. Protein extracts were loaded on 4 to 20% stain-free SDS-PAGE gels and subjected to electrophoresis before activation and transfer to a nitrocellulose membrane. Equal loading of samples was assessed qualitatively by 2,2,2-trichloroethanol visualization prior to blotting. Specific proteins were detected using the following primary antibody dilutions: anti-CtrA; 1:5,000 (kindly provided by MT Laub), anti-GcrA; 1:4,000 (72), anti-CcrM; 1:5,000 (73), anti-DnaA; 1:5,000 (74), anti-SciP; 1:2,000 (33), anti-Lon; 1:10,000 (kind gift from RT Sauer), anti-GroEL; 1:10,000 (8), anti-FzlA; 1:8,000 (kindly provided by ED Goley), and the commercially available anti-M2 1:1,000 (Sigma). Horseradish peroxidase (HRP)-conjugated secondary antibody raised against rabbit or mouse was used at a 1:5,000 dilution, and SuperSignal Femto West reagent was used for signal detection using a Licor Odyssey. Images were processed and quantified using Image Lab software (Bio-Rad).

### Microscopy and image analysis.

For cell length analysis, samples were fixed in 1% formaldehyde and spotted on 1% agarose pads. A final concentration of 2 μg/ml Hoechst 33258 was used to stain fixed cells by incubating 25 min in the dark prior to mounting. HADA staining was performed on cells prior to ethanol fixation as described previously (75). For live cell imaging, including all instances of fluorescent protein imaging, the microscope housing was heated to 30°C and live cells were spotted on 1% agar PYE pads containing xylose, glucose, or vanillate as necessary.

Imaging was performed on a Nikon Ti-Eclipse microscope equipped with a 100× objective and Zyla 4.2 Plus camera, and at least 10 independent frames of each sample were collected using Nikon Image Elements AR software. Image stacks were imported into Fiji and the background of fluorescent images was subtracted prior to segmentation using MicrobeJ (76). In all images, segmentation was manually checked prior to exporting data. Unless otherwise indicated, ANOVA analysis (including adjustment for multiple comparisons where necessary) was performed to derive statistical significance of morphological changes using GraphPad Prism 8 software.

### Flow cytometry.

Samples of C. crescentus cultures grown as indicated were fixed in a final concentration of 70% ethanol. Cells were pelleted and washed in 50 mM sodium citrate buffer containing 2 μg/ml RNase and incubated overnight at 50°C. A final concentration of 2.5 μg/ml SYTOX green was used to stain 1:10 dilutions of the RNA-digested samples immediately prior to processing by a BD Biosciences LSR-Fortessa flow cytometer. Data were analyzed and histograms prepared with FlowJo.

### Subcellular fractionation.

Isolation of the detergent-resistant insoluble fraction was adapted from reference 39 as follows. Log-phase cultures were harvested at the indicated time points or conditions and pelleted at 7,000 × *g* for 10 min at 4°C. Cells were washed once in buffer I (50 mM Tris-HCl [pH 8.0], 150 mM NaCl) and frozen at –80°C. Pellets were resuspended in buffer I supplemented with 12 U/ml benzonase and disrupted by sonication (10 cycles of 30s on, 30s off at 50% amplitude in a QSonica sonicator). Cellular debris was removed from the lysate by centrifugation at 5,000 × *g* for 10 min at 4°C and removing supernatant, and repeating this step. Protein concentration of lysate was determined by Nanodrop. To separate soluble and insoluble fractions, lysate was centrifuged at 20,000 × *g* for 20 min at 4°C. The insoluble fraction was washed in buffer I, resuspended by one cycle of sonication, and pelleted again, followed by incubation with 1% Triton X-100 for 1 h with regular vortexing. The insoluble fraction was pelleted again and washed an additional two times before resuspension in Laemmli buffer. Membrane fractions were prepared separately as described previously (77).

### RNA sequencing.

RNA of bacterial cultures was extracted using the RNeasy minikit (Qiagen). rRNA depletion, RNA library preparation, multiplexing, sequencing, and differential gene expression analysis was performed by GENEWIZ, South Plainfield, NJ. Sequencing was conducted using the Illumina HiSeq2500 platform in a 1 × 50-bp single-read configuration. Sequence reads were trimmed and, after trimming, sequence reads shorter than 30 nucleotides were discarded. Sequence reads were aligned to the reference genome Caulobacter crescentus NA1000 using the CLC Genomics Server program. Gene hit counts were measured and reads per kilobase million (RPKM) values were calculated. Fold changes in gene expression were determined by comparing gene expression between the GroESL depletion strain and the wild type. Gene expression data are available at the Gene Expression Omnibus repository: GSE162320.

### Mass spectrometry.

The insoluble, detergent-resistant fraction of cultures was harvested and prepared in biological duplicates according to the protocol described above. Protein digestion, TMT10plex isobaric labeling, and mass spectrometry were performed at the Clinical Proteomics Mass Spectrometry facility (Karolinska Institute, Karolinska University Hospital, Science for Life Laboratory). To determine differential abundance in the insoluble fractions, linear model analysis was performed as described previously (78). Only significantly changed protein abundances (*P* < 0.05) were considered for further analysis, as described in the text. For analysis of SCOP folds, fold identity was predicted from amino acid sequence using the SUPERFAMILY 2 database (79, 80).
