# Supplementary figures and images for: The Chaperonin GroESL Facilitates Caulobacter crescentus Cell Division by Supporting the Functions of the Z-Ring Regulators FtsA and FzlA
Source: mBio. 2021 May 4;12(3):e03564-20. doi: 10.1128/mBio.03564-20 (PMC8262945; doi:10.1128/mBio.03564-20)

Supplemental Figure 1

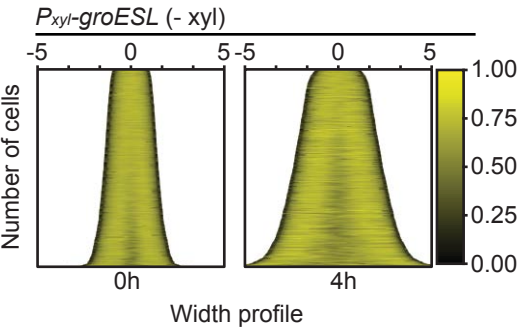

Supplement: FIG S1 [file mbio.03564-20-sf001.pdf]

Supplemental Figure 2

A

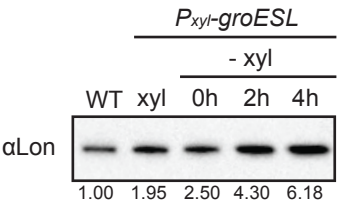

B

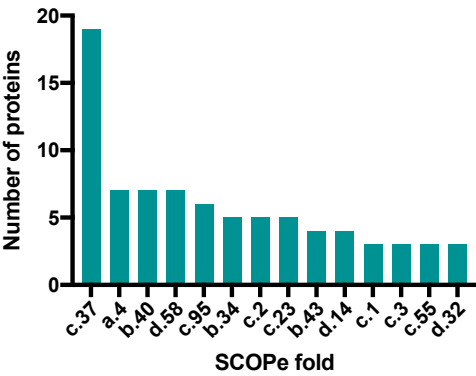

Supplement: FIG S2 [file mbio.03564-20-sf002.pdf]

Supplemental Figure 3

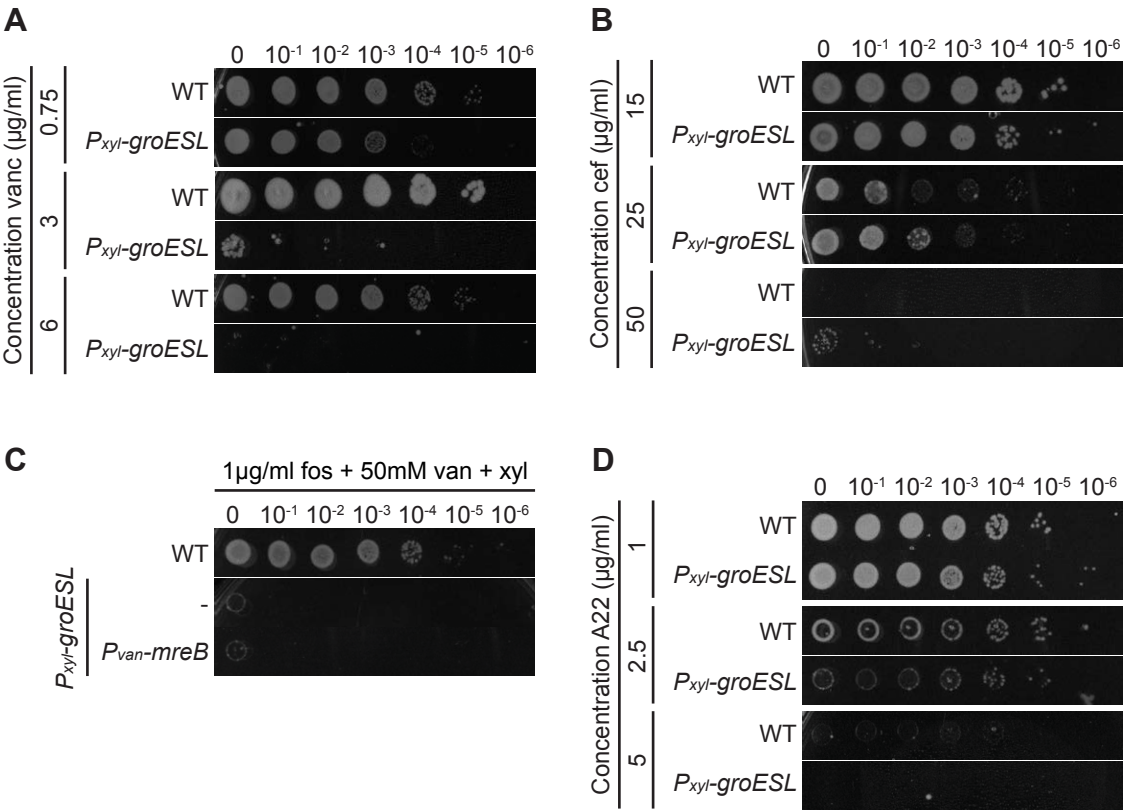

Supplement: FIG S3 [file mbio.03564-20-sf003.pdf]

**Supplemental Figure 4**

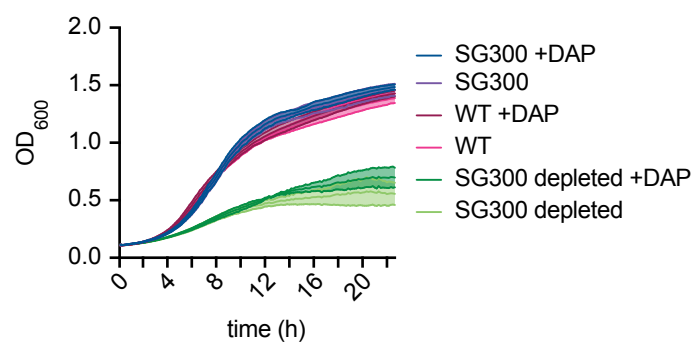

Supplement: FIG S4 [file mbio.03564-20-sf004.pdf]

**Supplemental Figure 5**

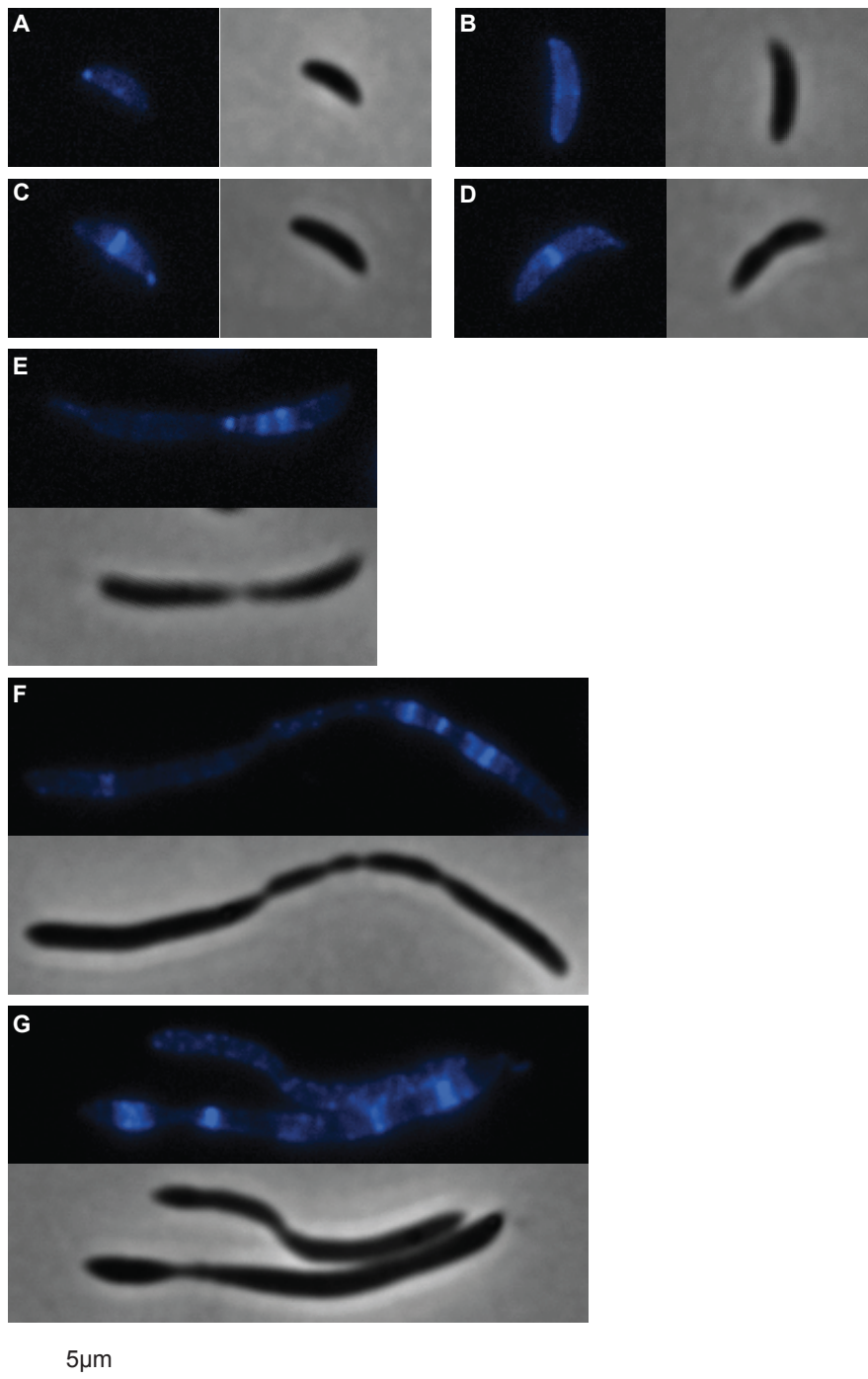

Supplement: FIG S5 [file mbio.03564-20-sf005.pdf]

Supplemental Figure 6

**A**

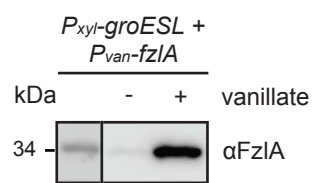

**B**

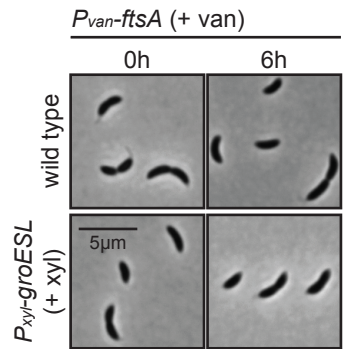

**C**

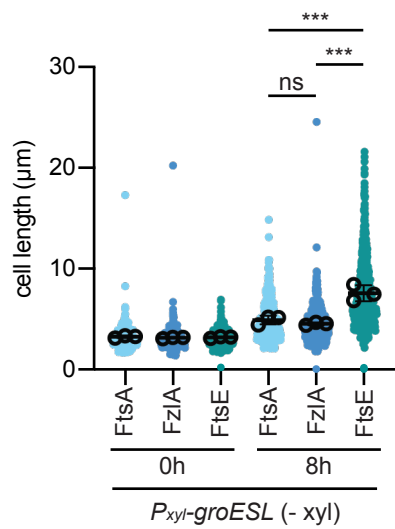

**D**

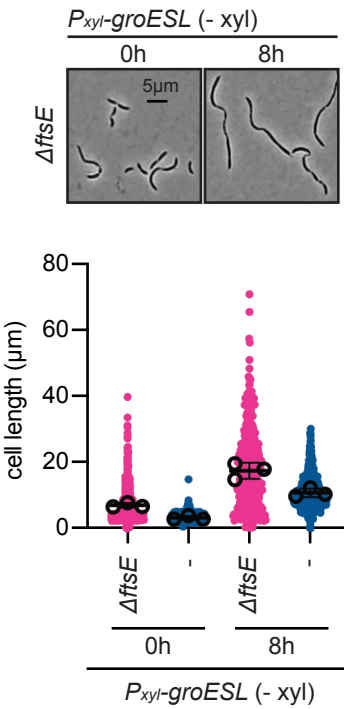

Supplement: FIG S6 [file mbio.03564-20-sf006.pdf]
